# Knowledge, Psychological Distress, and Medication Beliefs Among Tuberculosis Patients in Southwest China: A Mediation Analysis

**DOI:** 10.3390/healthcare14101298

**Published:** 2026-05-11

**Authors:** Yifei Zheng, Lingwei Dou, Chunnong Jike, Rujun Liao, Gang Yu, Ju Wang, Ruobing Wang, Yubing Wang, Ruili Bi, Rong Pei, Yuan Li

**Affiliations:** 1 School of Public Health, Chengdu University of Traditional Chinese Medicine, Chengdu 610075, China; 2 Warwick Business School, University of Warwick, Coventry CV4 7AL, UK; 3 Liangshan Prefecture Centre for Disease Control and Prevention, Xichang 615000, China; 4 Sichuan Provincial Center for Disease Control and Prevention, Chengdu 610041, China; 5 School of Public Administration, China University of Geosciences, Wuhan 430074, China

**Keywords:** path analysis, HIV-TB co-infection, health education, sociodemographic factors

## Abstract

**Background/Objectives**: Medication beliefs are important determinants of adherence in patients with chronic diseases. Failure to take medication threatens the successful treatment of tuberculosis. This research aimed to determine the associations of tuberculosis knowledge and psychological distress with medication beliefs in patients with tuberculosis from Southwest China. **Methods**: This study employed a cross-sectional design conducted in Liangshan Prefecture, Southwest China, from March 2024 to January 2025. The HIV-TB co-infection group comprised patients newly diagnosed with HIV-TB co-infection in 2024, while the TB mono-infection group comprised individuals randomly selected from those with tuberculosis mono-infection during the same period within the same region. Trained interviewers collected data through structured telephone questionnaires to assess medication beliefs, psychological distress, TB knowledge, and behavioral characteristics. Path analysis was used to examine the relationships between these variables. **Results**: Overall medication belief scores were low among participants. Beliefs varied significantly across clinical and sociodemographic factors, being relatively higher in groups such as patients with HIV co-infection, residents of formerly impoverished counties, individuals of Yi ethnicity, and those with primary education or less. Path analysis indicated that psychological distress partially mediated the relationship between TB knowledge and medication beliefs. The indirect effect was 0.014 (95% CI: 0.001, 0.033), accounting for 37.12% of the total effect of knowledge on beliefs, while the direct effect was non-significant. **Conclusions**: These findings underscore that both TB knowledge and psychological distress critically shape medication beliefs. Intervention strategies should therefore integrate evidence-based health education with targeted psychological support to strengthen treatment adherence and improve outcomes.

## 1. Introduction

According to the World Health Organization’s Global Tuberculosis Report 2025, an estimated 10.7 million people were newly diagnosed with tuberculosis (TB) worldwide in 2024, corresponding to an incidence rate of 131 cases per 100,000 population (95% confidence interval: 122–141 per 100,000). As one of the 30 high-TB-burden countries, China accounted for 6.5% of global TB incident cases in 2024 [1]. Substantial evidence demonstrates that medication adherence is a critical determinant of successful tuberculosis treatment and disease control [2,3,4,5]. Patient adherence to treatment regimens is influenced by multiple factors, among which individual beliefs and medication-related perceptions play pivotal roles in decision-making processes [6]. Consistent findings across studies identify medication beliefs as a core proximal predictor of adherence behavior [7,8,9,10,11,12]. Consequently, systematic assessment of patients’ medication beliefs enables the identification of individuals with insufficient perception of treatment necessity or heightened concerns regarding medications. This approach provides targeted intervention opportunities to optimize therapeutic outcomes.

**Figure 1:**
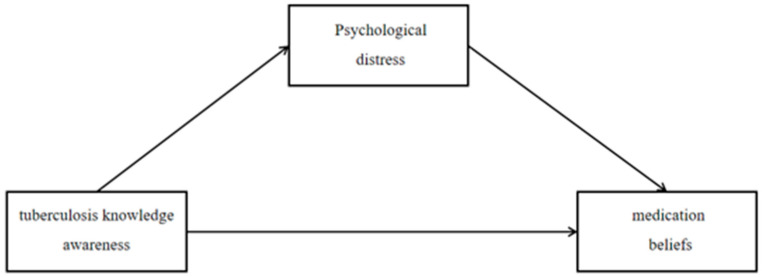
Pathway Framework for Analyzing Intermediation Effects.

The present study is guided primarily by the necessity–concerns framework [13], which posits that medication beliefs arise from the balance between perceived treatment necessity and concerns about potential harm. To complement this framework, we also draw on the theoretical logic of the Health Belief Model (HBM) [14], which suggests that cognitive factors and affective states may jointly influence health-related behavioral intentions. Previous research has established that tuberculosis knowledge influences patients’ understanding of their illness [15,16], while psychological distress is highly prevalent among tuberculosis patients and is associated with adverse treatment outcomes [17,18,19]. Awareness of illness influences psychological distress [20], which in turn may undermine patients’ positive expectations and beliefs regarding treatment efficacy and benefits [21], thereby diminishing medication beliefs. Although the core HBM constructs are not directly measured in this study, the model provides a useful conceptual backdrop for understanding the hypothesized sequential pathway from cognition to emotion to behavioral intention. Integrating these perspectives, the present study aims to examine the association of tuberculosis knowledge and psychological distress with medication beliefs among tuberculosis patients in Southwest China, testing a sequential pathway of “cognition → emotion → intention to engage in health-related behavior.” The following hypotheses are proposed (Figure 1 illustrates the hypothesized mediation framework):

**H1.**  *Tuberculosis knowledge is positively associated with medication beliefs.*

**H2.**  *Psychological distress is negatively associated with medication beliefs.*

**H3.**  *Tuberculosis knowledge is negatively associated with psychological distress.*

**H4.**  *Psychological distress mediates the association between tuberculosis knowledge and medication beliefs.*

This study employed structural equation modeling (SEM) to test the proposed mediation framework (Figure 1). By integrating the necessity–concerns framework with the cognitive–emotional pathway, the findings provide a theoretical foundation for developing targeted interventions to enhance medication beliefs among tuberculosis patients in Southwest China.

## 2. Materials and Methods

### 2.1. Study Participants

**Figure 2:**
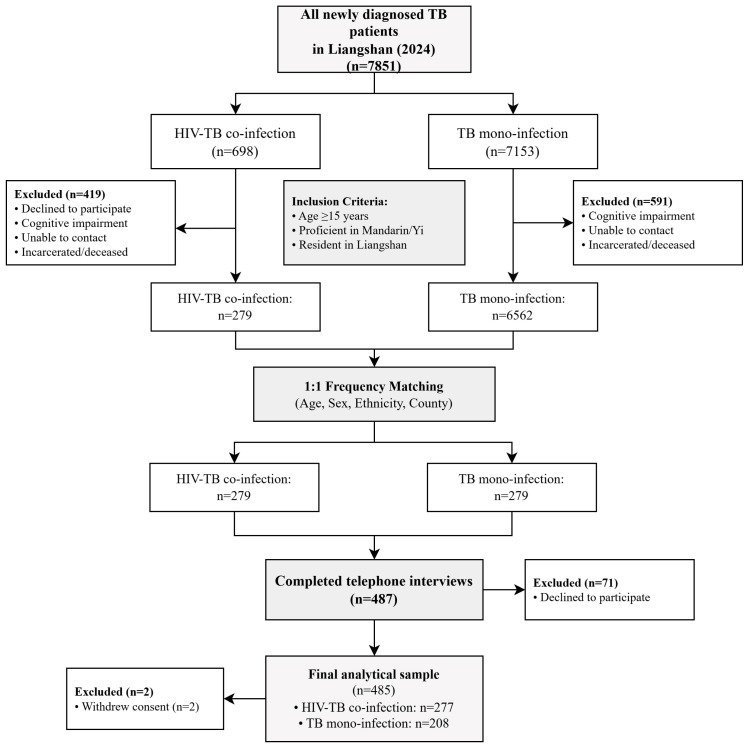
Flowchart for Inclusion and Exclusion of Research Subjects.

This cross-sectional study included two groups of participants: the HIV-TB co-infection group, comprising patients newly diagnosed with HIV-TB co-infection in 2024, and the TB mono-infection group, comprising individuals randomly selected from those with tuberculosis mono-infection during the same period and from the same geographic region. In the sampling stage, we matched participants between the two groups on age, gender, ethnicity, and county of residence to enhance group comparability and reduce potential confounding in subsequent descriptive comparisons. The primary analytical aim was to examine the mediation pathways among tuberculosis knowledge, psychological distress, and medication beliefs across the combined inclusion criteria: Aged ≥15 years; Proficient in Mandarin or the Yi language; Official residence registration in Liangshan Prefecture. Exclusion criteria: Patients with cognitive impairment; Incarcerated or deceased during the survey period; Declined to participate. The participant selection process is summarized in Figure 2.

### 2.2. Study Design and Procedures

This study was conducted in Liangshan Prefecture from March 2024 to January 2025. Participants were identified through the tuberculosis and HIV/AIDS Management Information System of Liangshan Prefecture. County/city health centers or follow-up physicians explained the study purpose and obtained informed consent. Informed consent was obtained from all participants. For participants aged 15–17, informed consent was obtained from both the minor and a parent or legal guardian, as approved by the ethics committee, and due to the telephone-based data collection method, consent was verbally obtained and documented by the interviewers. To address methodological considerations of telephone interviews, all enrolled participants were contacted by one of three trained interviewers who administered a structured questionnaire via telephone. Each participant was interviewed by a single interviewer to ensure consistency, and interviewer assignments were systematically rotated to minimize bias. This study was approved by the Ethics Committee of Chengdu University of TCM (Approval No. 2024KL-119-01).

### 2.3. Sample Size Calculation

The sample size was determined using the formula: n = Z^2^ × P(1 − P)/d^2^. Assuming a confidence level of 95% (Z = 1.96), an expected proportion (P) of 0.50, and a precision (d) of 0.05, the minimum required sample size was calculated as 384. After accounting for a 10% non-response rate, the target sample size was adjusted to 427, and the final sample size included 485 valid participants.

### 2.4. Questionnaire Development

The survey instrument was developed through literature review, expert consultation, and adaptation to local contexts. It comprised four modules:

- Demographics: Gender, age, education level, marital status, occupation, ethnicity
- Behavioral characteristics: Smoking, alcohol consumption, exercise habits
- Tuberculosis knowledge assessment: Seven questions to evaluate transmission, treatment, and prevention knowledge

#### 2.4.1. Validated Scales

- Psychological distress: 10-item Kessler Psychological Distress Scale (K10)
- Medication beliefs: Beliefs about Medicines Questionnaire (BMQ)

Scale Specifications: K10: Measures mental health status using 5-point Likert scales. Total score ranges 10–50, with higher scores indicating worse psychological distress [22]. Cronbach’s α = 0.87 in this study.

BMQ: Contains 10 items across two subscales: Necessity: 5 items (score 5–25); Concerns: 5 items (score 5–25). Total belief score = Necessity − Concerns (range: −20 to 20), with higher scores indicating stronger positive medication beliefs [23]. Cronbach’s α = 0.81.

Variable coding and directionality: tuberculosis knowledge was coded as the total number of correct responses (range: 0–7), with higher scores indicating better knowledge. Psychological distress was measured using the Kessler Psychological Distress Scale (K10), with total scores ranging from 10 to 50; higher scores indicate greater distress. Medication beliefs were calculated as the necessity score minus the concerns score (range: –20 to 20), such that higher positive scores reflect stronger positive beliefs about medication. All variables were treated as continuous observed variables in the path analysis.

#### 2.4.2. Definition and Assessment of Key Variables

Smoking and drinking status were defined as follows based on questionnaire responses: Current smoker: daily or occasional smoking in the past 6 months; Former smoker: lifetime smoking ≥ 100 cigarettes but none in the past 6 months; Never smoker: lifetime smoking < 100 cigarettes. Current drinker: drinking ≥ 1 time/week in the past 6 months; Former drinker: previously drank weekly but <1 time/week in the past 6 months; Never drinker: drinking < 1 time/week.

### 2.5. Quality Control

Quality control was rigorously implemented through standardized professional training for all survey staff to ensure mastery of research protocols, culturally adapted interviewing techniques, and effective communication strategies. To address language barriers, bilingual interviewers were specifically assigned for Yi-speaking participants. Additionally, a comprehensive quality control system included periodic on-site monitoring by the research steering committee, real-time technical support to maintain protocol adherence, and random callback verification of 15% completed questionnaires, ensuring strict compliance with the study design throughout data collection.

To establish cultural validity, the questionnaire was pilot tested with a small sample of Yi participants to assess comprehension and cultural appropriateness. Feedback from pilot testing informed minor wording adjustments to ensure that items were culturally relevant and clearly understood. The use of bilingual interviewers and culturally adapted interviewing techniques further supported the cultural appropriateness of data collection.

### 2.6. Statistical Analyses

Data were entered in duplicate using Epidata 3.1 software for consistency verification. General statistical analysis was performed using SPSS 26.0. As the quantitative data were not normally distributed, descriptive analysis presented categorical data as frequencies and proportions, while quantitative data were characterized by median values (interquartile range) [M (P25, P75)]. Mann–Whitney U or Kruskal–Wallis H tests were conducted for univariate analysis, with Spearman’s correlation coefficient employed for correlation analysis. While matching was used during participant selection to improve group comparability, the primary analytical goal was to examine the mediation pathways among tuberculosis knowledge, psychological distress, and medication beliefs across the full sample. Given this analytical focus, we pooled the two groups for the path analysis. Mediation effects were examined through structural equation modeling (SEM) in Amos 24.0. Path analysis within the SEM framework was used to estimate direct and indirect effects, with bootstrapping applied to assess the significance of the mediation pathways. All tests were two-sided with statistical significance set at α = 0.05.

## 3. Results

### 3.1. Sociodemographic Characteristics of Tuberculosis Patients in Liangshan

Among 487 questionnaires collected, 485 were valid for analysis (valid response rate: 99.59%). Key characteristics of participants include: 277 (57.11%) with HIV-TB co-infection; 339 males (69.90%); and a balanced age distribution across groups. The cohort was predominantly of Yi ethnicity (87.84%). Married/cohabitating individuals comprised 55.46% of the sample. 220 (45.36%) were unemployed. Regarding medication beliefs, the median scores for the Necessity and Concerns subscales were 20 (18, 22) and 18 (17, 20), respectively. The BMQ Score showed a relatively low median of 1 (0, 3). HIV-TB co-infection, residing in formerly impoverished counties [24], males, aged 30–39 years, Yi ethnicity, primary education or below, farmers and workers group demonstrated higher medication beliefs than other groups. Table 1 summarizes the detailed sociodemographic characteristics of tuberculosis patients in Liangshan Prefecture.

### 3.2. Behavioral Characteristics of Tuberculosis Patients in Liangshan

Study findings revealed a relatively high smoking prevalence (49.90%) and a low current alcohol consumption rate (10.72%) among tuberculosis patients in Liangshan. Regarding physical activity, 46.60% maintained regular exercise, while 29.90% reported insufficient physical activity. Among all the participants, the current smoker group exhibits higher medication beliefs scores than other groups. Table 2 shows the behavioral characteristics of the respondents included in the study.

### 3.3. Partial Correlation Analysis of Tuberculosis Knowledge, Psychological Distress, and Medication Beliefs

The partial correlation analysis among tuberculosis knowledge, psychological distress, and medication beliefs revealed significant associations. Specifically, tuberculosis knowledge was negatively correlated with both psychological distress and medication beliefs (r < 0, *p* < 0.01). Conversely, a positive correlation was observed between psychological distress and medication beliefs (r = 0.12, *p* < 0.01). Detailed results are presented in Table 3.

### 3.4. Mediating Role of Psychological Distress Between Tuberculosis Knowledge and Medication Beliefs Among Tuberculosis Patients in Liangshan Prefecture

**Figure 3:**
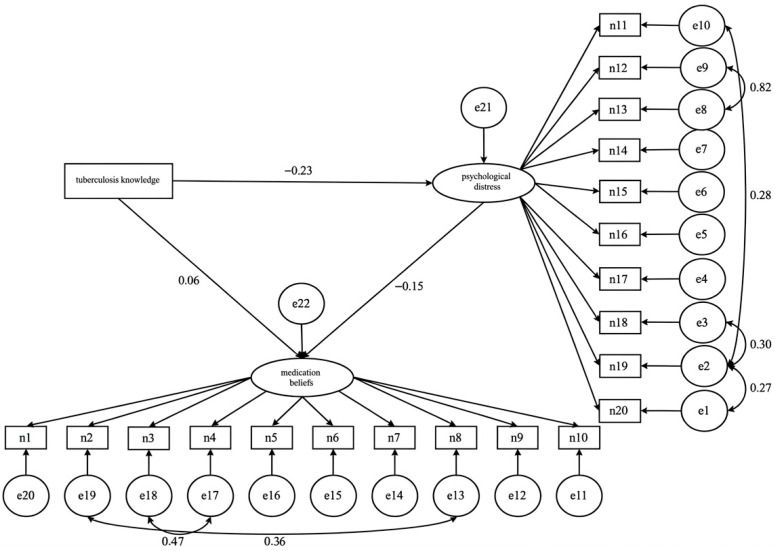
The Mediating Role of Psychological Distress Between Tuberculosis Knowledge and Medication Beliefs.

Using Amos 24.0 with bootstrapping, we examined whether psychological distress mediated the relationship between tuberculosis knowledge and medication beliefs (Table 4, Figure 3). The structural equation model demonstrated modest fit(χ^2^/DF = 4.135, RMSEA = 0.080, GFI = 0.866, AGFI = 0.829, CFI = 0.881, TLI = 0.862) [25,26,27,28]. Given the modest model fit, the following mediation results should be interpreted as indicative rather than definitive. As shown in Table 4, the direct effect of tuberculosis knowledge on medication beliefs was not statistically significant (β = 0.023, *p* = 0.199), whereas the indirect effect via psychological distress was significant (β = 0.014, 95% CI: 0.001, 0.033). The indirect effect accounted for 37.12% of the total effect. These findings suggest that the association between TB knowledge and medication beliefs was statistically explained by psychological distress in the proposed model. However, given the modest model fit, this mediation finding should be considered preliminary and interpreted with caution. The indirect effect was modest, and the direct effect remained non-significant.

## 4. Discussion

This study found that psychological distress played a mediating role in the relationship between tuberculosis knowledge and medication beliefs among tuberculosis patients, with the association operating primarily through this pathway in the proposed model. Higher tuberculosis knowledge levels were found to correlate with reduced psychological distress and greater medication beliefs. Previous studies have substantiated that heightened comprehension of maladaptive illness perceptions exhibited a dose-dependent relationship with the exacerbation of affective symptomatology, particularly depressive and anxiety disorders [29,30]. Other studies confirmed that elevated psychological distress adversely impacted medication beliefs through dual pathways: compromising perceived therapeutic necessity while amplifying medication-related concerns, collectively diminishing composite medication beliefs [31,32,33].

Our findings revealed that overall medication belief scores were only weakly positive among TB patients in Southwest China. A closer examination of the BMQ subscales indicated that high Necessity scores were partially offset by high Concern scores, reflecting a state of ambivalence: patients recognized a strong perceived need for treatment to maintain their health, yet this was coupled with substantial apprehension regarding potential side effects and the long-term toxicity of anti-TB drugs. This pattern aligns with the necessity–concerns framework, which emphasizes that medication beliefs arise from the balance between perceived treatment necessity and concerns about potential harm [13]. Healthcare providers should therefore not only reinforce the necessity of treatment but, more importantly, proactively address specific patient concerns regarding drug safety to tilt the balance toward more robust positive beliefs [34]. Furthermore, the psychological distress reported by patients may stem not only from the burden of the disease itself but also from the neuropsychiatric effects of anti-TB medications. Certain first-line anti-TB drugs, such as isoniazid, are known to have central nervous system side effects that can induce or exacerbate anxiety, depression, and psychosis [35,36]. These pharmacological effects may confound or amplify the observed relationship between TB knowledge and psychological distress, independent of patients’ cognitive understanding of their illness. Therefore, interventions aimed at reducing psychological distress and strengthening medication beliefs should consider both the psychosocial and the pharmacogenic contributors to patients’ emotional state, integrating mental health support as a routine component of TB care.

Contrary to our hypothesis, the bivariate correlation showed a negative association between TB knowledge and medication beliefs (r = −0.15, *p* < 0.01). However, the subsequent path analysis revealed a positive indirect effect via psychological distress and a non-significant direct effect. This pattern suggests a suppression effect [37,38]. The negative correlation may be driven by the emotional impact of knowledge. That is, while TB knowledge itself may not directly harm medication beliefs, health education that inadvertently increases psychological distress could lead to less positive beliefs. Therefore, the manner in which knowledge is communicated, specifically managing emotional responses, may be as important as the knowledge content itself. These results are consistent with the view that knowledge itself may not be detrimental to medication beliefs; rather, when health education is associated with increased psychological distress—such as anxiety or fear—it may correlate with less positive beliefs. Thus, enhancing medication beliefs requires not only improving TB knowledge but also carefully managing the emotional responses elicited during health education.

The hypothesis proposed in the study was not fully supported by the findings, and in the proposed model, the direct pathway from tuberculosis knowledge to medication beliefs was not statistically significant, although this does not preclude the existence of a direct relationship that may be context-dependent or masked by other unmeasured factors. Extant international studies demonstrated that respondents with limited disease knowledge exhibited heightened concerns regarding medication misuse and adverse effects [34], thereby reducing their medication beliefs. This discrepancy may be explained by the distinct indigenous disease perceptions and health belief frameworks among tuberculosis patients in Liangshan Prefecture, Sichuan Province, China. Their comprehension of tuberculosis likely extended beyond biomedical knowledge, syncretizing ethnic-cultural spirituality rather than being solely based on biomedical knowledge. which may contribute to the weaker association between knowledge metrics and medication beliefs observed in this cohort.

This study found that HIV-TB co-infected patients had significantly higher medication belief scores than TB mono-infected patients (*p* < 0.01). First, co-infected individuals typically received more intensive adherence education during clinical consultations. In contrast, some TB mono-infected patients might report weaker perceived medication necessity due to factors such as inadequate disease awareness [39]. Second, the implementation of the “Phase II Campaign (2021–2025) Against HIV/AIDS and Major Infectious Diseases in Liangshan Prefecture” [40] had substantially improved antiretroviral therapy (ART) coverage and success rates in this region [41]. This progress likely enhanced co-infected patients’ confidence in treatment efficacy, thereby strengthening their overall medication beliefs. Furthermore, existing evidence indicated that greater disease severity reinforced patients’ perceived necessity of medication [42]. As HIV-TB co-infection represented a more severe disease status, this mechanism might have further elevated medication beliefs in this population. Consequently, intensified education on medication adherence for TB mono-infected patients was urgently needed to enhance their medication beliefs and increase treatment success rates.

The results showed that both Yi ethnicity and lower educational attainment were associated with stronger medication beliefs. Previous studies had suggested that populations with limited education were geographically concentrated in Yi-inhabited areas [43]. For such populations, healthcare providers have been able to more effectively reinforce the perceived necessity of taking medicine through simplified health education and drug guidance, directly emphasizing the urgency and importance of TB treatment. Consistent with these findings, existing research has reported associations between both Yi ethnicity and low education with higher medication adherence [44,45], which is conceptually aligned with the core observation of this study regarding medication beliefs in these groups.

Age was identified as a significant predictor of medication beliefs. Consistent with prior research, the perceived necessity of medication strengthened with advancing age, particularly among older patient cohorts [31]. Individuals aged 15–30 years exhibited lower medication beliefs, potentially attributable to the higher prevalence of chronic conditions and subsequent polypharmacy treatment among older age groups [46]. Research on chronic conditions has shown that a majority of patients perceive their currently prescribed medications as essential for health management [47].

Our findings revealed higher medication belief scores among smokers. existing evidence demonstrated that smoking is associated not only with severe forms of tuberculosis in terms of sequelae but also with poor treatment outcomes, such as recurrence and death [48]. Patients typically exhibit pronounced respiratory symptoms such as a chronic cough [49]. Furthermore, extant research highlighted that tuberculosis exhibited a strong socioeconomic patterning, predominantly concentrated in resource-deprived regions. Therefore, we hypothesized that smokers’ strong motivation for a cure stems from a biological imperative to eliminate infectiousness and alleviate respiratory symptoms. This reinforces their recognition of the necessity of medication and the urgency of treatment, constituting a key pathway for this group to develop a high level of medication beliefs.

Notably, this pattern was more prominent among males, consistent with the finding that men had stronger medication beliefs. Behaviorally, males consistently have higher smoking prevalence than females across geographic regions [50,51,52,53]. Elevated smoking prevalence is associated with greater susceptibility to exacerbated tuberculosis manifestations, such as asthenia and persistent cough. From a sociocultural perspective, men were frequently positioned as primary household breadwinners. Tuberculosis-associated work capacity impairment may affect their economic contribution and be associated with greater psychological distress stemming from perceived inability to fulfill familial obligations. This dual anxiety may contribute to a stronger conviction in expedited recovery. Consequently, the observed elevation in medication beliefs among workers and farmers as occupational groups could be explained through the interplay between smoking behaviors and sociocontextual determinants. First, significantly higher smoking prevalence among industrial and agricultural workers versus other occupational groups [54], increased their vulnerability to smoking-aggravated tuberculosis manifestations. Second, Second, in these physically demanding occupations, respiratory symptoms… may be more disruptive to work capacity, and the risk of income disruption may be linked to higher perceived therapeutic necessity. Concurrently, as populations with constrained economic resources, workers and farmers represent a group that aligns with tuberculosis’ socioeconomic epidemiology [49].

Despite the valuable findings, this study has several limitations. Because we used a cross-sectional survey design, we cannot draw causal conclusions about the relationships between the variables. Another issue is that although we used matching at the sampling stage to improve group comparability, our main mediation analysis was done on the pooled sample without including the matching variables as covariates. We made this choice because we wanted to examine general mediation mechanisms across the whole sample, and the sample size was too small for more complex stratified or conditional analyses. Future studies with larger samples could build on these findings using methods that properly account for matched designs. Moreover, key potential confounders—such as socioeconomic status, treatment phase, tuberculosis-related stigma, and comorbid chronic diseases—were not adjusted for in the mediation model, partly due to the same sample size constraints. As a result, we cannot rule out the possibility that unmeasured factors may have influenced the observed associations. In addition, the study was conducted only in Liangshan Yi Autonomous Prefecture, an area with a unique cultural context and a high tuberculosis burden, so the findings may not generalize to other populations and broader validation is needed. Data were collected by telephone, which can introduce measurement bias such as social desirability bias and limited depth in responses, and self-reported data also come with limitations like recall inaccuracy. Future research could strengthen validity by combining objective measures, such as pill counting, or using qualitative approaches to add depth. Finally, the structural equation model showed only modest fit, meaning the proposed mediation structure may not fully capture the patterns in the data. So even though the indirect effect was statistically significant, the model should be seen as offering preliminary support for the mediation hypothesis rather than strong confirmation.

## 5. Conclusions

This study provides preliminary evidence that addressing psychological distress, alongside improving tuberculosis knowledge, may be important for fostering better medication beliefs in tuberculosis patients. Future interventions should focus on enhancing patients’ tuberculosis knowledge through scientifically grounded health education, while alleviating psychological distress to strengthen medication beliefs in treatment regimens, thereby achieving successful cure of tuberculosis.

## Figures and Tables

**Table 1:** General Conditions of Tuberculosis Patients in Liangshan.

Group	n (%)	Necessity Median [P25, P75]	Concerns Median [P25, P75]	BMQ Scores (Median [P25, P75])	Statistic (Z/H)	*p*-Value *
**Infection Status**						
TB mono-infection	208 (42.89%)	20 (19, 20)	18 (18, 20)	1 (0, 2)	−3.67	<0.001
HIV-TB co-infection	277 (57.11%)	21 (18, 24)	19 (16, 21)	2 (0, 4)		
**Region**						
Formerly impoverished county	149 (30.72%)	18 (16, 20)	17 (14, 18)	2 (0, 3)	8.26	0.006
Non-impoverished county	336 (69.28%)	19 (15, 20)	18 (14, 19)	1 (−1, 2)		
**Gender**						
Male	339 (69.90%)	20 (18, 23)	19 (17, 20)	2 (0, 3)	−2.41	0.016
Female	146 (30.10%)	20 (18, 20)	18 (18, 20)	1 (−0.25, 2)		
**Age Group (years)**						
15–29	100 (20.62%)	20 (19, 20)	20 (18, 21)	0 (0, 2)	9.12	0.028
30–39	132 (27.22%)	20 (18, 24)	19 (17, 20.75)	2 (0, 3.75)		
40–49	126 (25.98%)	20 (18, 24)	18 (15.75, 21)	1 (−1, 4)		
≥50	127 (26.19%)	20 (18, 21)	18 (18, 19)	2 (0, 2)		
**Ethnicity**						
Yi	426 (87.84%)	20 (18, 23)	18 (17, 20)	2 (0, 3)	−2.85	0.004
Other	59 (12.16%)	20 (17, 20)	19 (18, 20)	0 (−1, 2)		
**Education Level**						
Illiterate/Semi-illiterate	212 (43.71%)	20 (18, 23)	18 (17, 20)	2 (0, 3)	20.89	<0.001
Primary school	141 (29.07%)	20 (18, 24)	18 (17, 20)	2 (0, 3)		
Junior high	82 (16.91%)	20 (18, 21)	19 (18, 20)	0.5 (0, 2)		
High school/Technical	22 (4.54%)	20 (20, 22.25)	20 (19, 21)	0 (−1, 1.25)		
College+	28 (5.77%)	20 (18, 20)	20.50 (17.25, 21.75)	−0.5 (−1, 1)		
**Marital Status**						
Single	88 (18.14%)	20 (18.25, 20)	19 (18, 20.75)	0 (−0.75, 2)	5.66	0.129
Married/Cohabitating	269 (55.46%)	20 (18, 23)	19 (18, 20)	2 (0, 3)		
Divorced/Separated	79 (16.29%)	20 (18, 24)	18 (17, 20)	1 (0, 3)		
Widowed	49 (10.10)	20 (17.50, 21)	18 (16, 19)	2 (−0.5, 2.5)		
**Occupation**						
Farmer	125 (25.77%)	20 (18, 22.50)	18 (16, 20)	2 (0, 4)	19.10	<0.001
Worker	63 (12.99%)	20 (19, 24)	19 (17, 21)	2 (0, 4)		
Unemployed	220 (45.36%)	20 (18, 22)	18 (18, 20)	1 (0, 2)		
Other	77 (15.88%)	20 (20, 20)	20 (18, 21)	0 (−1, 2)		

* *p*-values are derived from comparisons of the total BMQ score; subscale scores are presented for descriptive purposes only.

**Table 2:** Behavioral Characteristics of Tuberculosis Patients in Liangshan.

Group	n (%)	Necessity Median [P25, P75]	Concerns Median [P25, P75]	BMQ Scores (Median [P25, P75])	Statistic (Z/H)	*p*-Value
**Smoking Status**						
Current Smoker	242 (49.90%)	20 (18, 24)	19 (17, 21)	2 (0, 3.25)	10.40	0.006
Never Smoked	181 (37.32%)	20 (18, 20)	18 (18, 20)	1 (0, 2)		
Quit Smoking	62 (12.78%)	20 (19, 21.25)	18 (15.75, 20)	2 (0, 4)		
**Alcohol Use**						
Current Drinker	52 (10.72%)	19 (17, 21.50)	18 (16, 20)	1 (−1, 4)	2.37	0.307
Never Drank	257 (52.99%)	20 (19, 21)	19 (18, 20)	1 (0, 2)		
Quit Drinking	176 (36.29%)	20 (18, 23)	18 (17, 20)	2 (0, 3)		
**Exercise Frequency**						
Regularly	226 (46.60%)	20 (18, 23)	19 (18, 20)	2 (0, 3)	0.65	0.722
Occasionally	114 (23.51%)	20 (19, 21)	19 (18, 20)	1 (0, 2)		
Never	145 (29.90)	20 (18, 21)	18 (16.50, 20)	1 (−1, 3)		

Note: *p*-values are derived from comparisons of the total BMQ score; subscale scores are presented for descriptive purposes only.

**Table 3:** Partial Correlation Coefficients (r) Among Tuberculosis Knowledge, Psychological Distress, and Medication Beliefs in Tuberculosis Patients from Liangshan Prefecture.

Variable	Tuberculosis Knowledge	Psychological Distress	Medication Beliefs
Tuberculosis Knowledge	1.00	−0.20 **	−0.15 **
Psychological Distress	−0.20 **	1.00	0.12 **
Medication Beliefs	−0.15 **	0.12 **	1.00

Note. ** *p* < 0.01.

**Table 4:** The Mediating Role of Psychological Distress Between Tuberculosis Knowledge and Medication Beliefs Among Tuberculosis Patients in Liangshan Prefecture.

Path	Effect Size	*p*-Value	Bootstrap 95% CI	Proportion
Direct Effect	0.023	0.199	(−0.013, 0.062)	62.88%
Indirect Effect	0.014	0.030	(0.001, 0.033)	37.12%
Total Effect	0.037	0.044	(0.001, 0.076)	

## Data Availability

The datasets generated and/or analysed during the current study are not publicly available due to privacy or ethical restrictions, but are available from the corresponding author on reasonable request.
